# Use of Video Consultation Between 2017 and 2020 in Outpatient Medical Care in Germany and Characteristics of Their User Groups: Analysis of Claims Data

**DOI:** 10.2196/60170

**Published:** 2025-03-14

**Authors:** Theresa Hüer, Anke Walendzik, Lara Kleinschmidt, Klemens Höfer, Beatrice Nauendorf, Juliane Malsch, Matthias Brittner, Paul Brandenburg, André Aeustergerling, Udo Schneider, Anja Wadeck, Sebastian Liersch, Stephanie Sehlen, Katharina Schwarze, Jürgen Wasem

**Affiliations:** 1 Institute for Health Care Management and Research University of Duisburg-Essen Essen Germany; 2 Kassenärztliche Vereinigung Berlin Berlin Germany; 3 Kassenärztliche Vereinigung Westfalen-Lippe Dortmund Germany; 4 Kassenärztliche Vereinigung Schleswig-Holstein Bad Segeberg Germany; 5 Kassenärztliche Vereinigung Mecklenburg-Vorpommern Schwerin Germany; 6 Techniker Krankenkasse Hamburg Germany; 7 AOK Nordost Potsdam Germany; 8 AOK NordWest Dortmund Germany

**Keywords:** video consultation, outpatient medical care, user groups, claims data analysis, Germany, physician, psychotherapist, sociodemographic, healthcare, digital health, digital consultation, telehealth, telemonitoring, telemedicine

## Abstract

**Background:**

Supplementing outpatient medical care with the use of video consultations could, among other benefits, improve access, especially in structurally disadvantaged areas.

**Objective:**

This claims data analysis, carried out as part of the German research project “Preference-based use of video consultation in urban and rural regions,” aimed to analyze the use of video consultations and the characteristics of its user groups.

**Methods:**

Claims data from 3 Statutory Health Insurance Funds (SHIFs) and 4 Associations of Statutory Health Insurance Physicians (ASHIPs) from the period April 2017 to the end of 2020 were used. Data from a sample of about 6.1 million insured and 33,100 physicians and psychotherapists were analyzed. In addition to data on the use of video consultations, patient data on sociodemographic characteristics, diagnoses, and place of residence were included. To analyze the physicians’ perspectives, specialty groups, demographic characteristics, and the type of practice location were also included. In consideration of the principles of data economy and the fact that data analysis represents merely a preliminary phase within the broader project, the SHIFs and ASHIPs transmitted aggregated data (cross-tabulations per subgroup analysis) to the evaluator. For this reason, the analyses were constrained to a comparison of video consultation users versus nonusers, differentiated according to the aforementioned subgroups. Furthermore, the association between place of residence or type of region of the practice location and the use of video consultation was examined. A significance level of *P*<.05 was set for chi-square tests.

**Results:**

From 2017 to 2019, almost no video consultations were used in outpatient care in the German health care system. Although this changed considerably in relative terms with the start of the COVID-19 pandemic (but still at a very low absolute level), there was also a clear decline in the use of video consultations as the number of infections flattened out. Physicians working in psychotherapy and psychological psychotherapists used video consultations with around 16% (44,808/282,530) of their treatment cases in the second quarter of 2020, followed by psychotherapists using video consultations for children (10,828/113,293, 10%). Although the absolute number of treatment cases with video consultations among general practitioners was very high compared with other specialist groups, their share of video consultations in all treatment cases was very low at 0.3% (29,600/9,837,118). Younger age groups and those located in urban areas used video consultations more frequently; this applies to both patients (age groups: χ^2^_7_=9903.2, *P*<.001; region types: χ^2^_2_=3746.2, *P*<.001) and service providers (age groups: χ^2^_3_=11,338.2, *P*<.001; region types: χ^2^_2_=8474.1, *P*<.001).

**Conclusions:**

The current use of video consultations is below its potential in terms of scope and user groups. The widespread and lasting use of video consultations will only succeed if the potential user groups accept this form of service provision and recognize its advantages. Further analyses (both qualitative, such as focus group discussions, and quantitative, such as preference surveys) should therefore investigate the preferences of user groups for the use of video consultations.

**International Registered Report Identifier (IRRID):**

RR2-10.2196/50932

## Introduction

The implementation of video consultations in the German Social Health Insurance (SHI) system in April 2017 was intended to establish a basis for providing outpatient medical services regardless of location and thus also in structurally disadvantaged regions in Germany. Older people and those with limited mobility were to be given easier access to medical services. Video consultations can be used in a variety of ways and therefore offer potential benefits for different patient groups. Particularly in the case of long travel distances to the nearest (specialist) physician, for follow-up appointments, or for the provision of repeated prescriptions, video consultations can be a useful tool, and the insured do not have to visit a doctor's office for every appointment. In the field of psychotherapy, therapeutic or certain diagnostic sessions can also take place in the form of video consultations [1-3].

In the beginning, video consultations in Germany could only be provided by a few specialist groups and only for selected indications and follow-up appointments. In the second quarter of 2019, these restrictions were mostly abolished, and the assessment of the appropriateness of a diagnosis or treatment via video consultation was the responsibility of the respective physician. The remuneration for video consultations in outpatient medical care within the German SHI system is primarily based on age-differentiated quarterly flat rates. These rates cover the entire medical care of a patient, not just video consultations, provided by the attending physician during the specified period. If a patient is treated exclusively via video in a quarter and there are no other personal physician-patient contacts, the quarterly flat rate is reduced by a discount specific to the specialist group. In addition to the quarterly flat rates per patient, there are supplementary fee schedule positions (FSPs) that compensate for the additional technical effort (€4.60 [US $4.77] per video consultation, FSP 01450) and the digital authentication of an unknown patient in video consultations (€1.15 [US $1.19] per video consultation with a previously unknown patient, FSP 01444). From October 2019 to the end of September 2021, there was also an incentive grant (at that time approximately €10 [US $10.36] per video consultation for a maximum of 50 video consultations per physician) to stimulate the use of video consultations [1].

Despite the potential of video consultations for different user groups (patients as well as physicians and psychiatrists), financing by the German SHI system since 2017, and support measures such as incentive payments, video consultations do not yet appear to have established themselves as an integral part of outpatient medical care. In order to investigate the underlying obstacles, it is important to examine the actual use of video consultations on a solid data basis in detail in order to identify suitable groups and occasions for the development of measures to promote video consultations.

The German committee for negotiating outpatient medical remuneration in the SHI system (“Bewertungsausschuss Ärzte”) published a report on the use of video consultations in 2018 and in an updated form in 2022. These reports show the development of the billing frequency of video consultations in outpatient medical care within the SHI system for the data period April 2017 to the end of 2021, differentiated according to the specialist groups as well as the age groups and gender of the insured [1,4]. In 2022, Gensorowsky et al [5] also published an article on the use of video consultations in Germany based on data from a nationwide health insurance fund, which also covered the billing frequency of video consultations for the period April 2017, but only up to the end of 2018. The analysis was differentiated according to the specialist groups of the physicians and the age groups and type of region in which insured persons who used video consultations live [5]. However, Gensorowsky et al [5] only examined the period up to the end of 2018 and were therefore unable to account for the effects of the COVID-19 pandemic. A more recent 2023 study by Petrick and Kreuzenbeck [6] considered data through mid-2020 but was based on data from only one Social Health Insurance Fund (SHIF) and therefore did not include information from the perspective of physicians as well as psychotherapists. Thus, recent studies are needed to investigate the characteristics of insured persons using the service. Additionally, it would be interesting to explore the specialist groups that offer video consultations. It is not possible to analyze the characteristics of physicians and psychotherapists (eg, provision of video consultations by age group, gender, region of practice location) using data from SHIFs. This requires data from the Associations of Statutory Health Insurance Physicians (ASHIPs).

Against this background, this article dealt in particular with closing the aforementioned gaps in the evidence by analyzing not only claims data from SHIFs but also data from ASHIPs. The data period April 2017 to the end of 2020 was included in order to take the effects of the COVID-19 pandemic into consideration, a relevant extension of the observation period compared with the paper by Gensorowsky et al [5], which ended in 2018. In addition to the analysis of the frequency of video consultations, detailed characteristics of their user groups (users and insured persons and providers and physicians and psychotherapists) will also be examined. The age and gender distributions, employment status, and residence or type of region will be compared between users and nonusers of video consultations. In the case of service providers, specialty groups as well as their age and gender distributions, type of operation and employment, and the type of region of the practice location were considered. In particular, a detailed analysis of the association between place of residence or type of region of the practice location and the use of video consultations represents a significant contribution to the literature.

This claims data analysis was conducted as part of the German research project “Preference-based use of video consultations in urban and rural regions,” which is funded by the Innovation Fund of the German Federal Joint Committee (funding number 01VSF20011). The research project addresses the question of how video consultations should be designed to enhance the quality of outpatient medical care while considering the preferences of insured individuals, physicians, and psychotherapists. A study protocol has been published elsewhere [7].

The objective of this data analysis was to examine the use of video consultations in Germany, with a particular emphasis on the impact of the COVID-19 pandemic, and to discern salient disparities between patient and physician subgroups. Our objective was to ascertain the prevalence of dependencies according to the following sociodemographic characteristics of insured persons: age group, gender, employment status, and place of residence. In addition, we wanted to investigate how video consultations were integrated into the overall treatment of a patient during the quarter; was this done exclusively via video, or was the video consultation only used as a supplement? For physicians and psychotherapists, the aim was to identify dependencies between video consultation use and the following variables: age group, gender, specialist group, practice type, and practice location.

## Methods

### Data

In this analysis, anonymized, aggregated data from 3 SHIFs (Techniker Krankenkasse, AOK Nordost, AOK NordWest) as well as 4 ASHIPs were used to reflect both the perspective of the insured or patients and that of the physicians and psychotherapists. Data from April 2017 to the end of 2020 from insured and physicians as well as psychotherapists who lived or practiced in 1 of 4 (of a total of 17) German regions (“Westphalia-Lippe,” “Mecklenburg-Western Pomerania,” “Schleswig-Holstein,” and “Berlin”) were analyzed. These regions were selected because they represent both rural and urban districts. They were classified as rural or urban districts using the interactive web application “INKAR - Indicators and Maps of Spatial and Urban Development” from the German Federal Institute for Research on Building, Urban Affairs and Spatial Development. Among other things, the settlement structure of the district is considered a combination of population density and the proportion of settlement area.

Aggregated data from about 6.1 million insured living in the 4 selected regions were analyzed. This corresponds to a share of approximately 8.4% of all SHI-insured persons in Germany (annual average in 2020=73,274,131) [8]. In addition to data on the use of video consultations, patient data on sociodemographic characteristics and diagnostic information were included. Of the 6.1 million insured persons included in the analysis, approximately 4.6 million (approximately 74%) had at least one outpatient treatment case on average across all included quarters (“users of outpatient services”). The group of outpatient service users is an important group in the analysis, as only they were faced with the actual decision of whether to contact a physician in person or via video. Table 1 summarizes the details of the SHIF data used and the characteristics of the insured who were included.

Table 2 shows the key information from the ASHIP data and the characteristics of the approximate 33,000 physicians and psychotherapists included. This corresponds to 18.4% (33,000/180,581 in 2020) of all SHI-accredited physicians and psychotherapists in Germany [9].

**Table 1:** Social Health Insurance Fund (SHIF) data.

Data type			Data
Source			3 SHIFs: Techniker Krankenkasse, AOK Nordost, AOK NordWest
Regions			4 of 17 regions in Germany: Berlin, Schleswig-Holstein, Mecklenburg-Western Pomerania, Westphalia-Lippe
Time period			Q2^a^ 2017 until the end of 2020
Exclusion criteria			1. Insured living abroad, 2. Insured in employee health insurance in the respective SHIF, 3. Insured who refused the use of their data for research purposes
**Insured persons (Q2 2017-Q4^b^ 2020)**			
	Minimum (Q4 2017), n	5,915,199	
	Maximum (Q2 2020), n	6,318,851	
	Average	6,131,573	
**Insured persons with at least one treatment case (Q2 2017-Q4 2020), n (%)**			
	Minimum (Q2 2020)	4,277,149 (67.7)	
	Maximum (Q1^c^ 2020)	4,761,242 (76.7)	
	Average	4,550,963 (74.2)	
**Gender** **(Q2 2020;** **n=4,277,148), n (%)**			
	Female (including diverse)	2,369,554 (55.4)	
	Male	1,907,594 (44.6)	
**Age (years;** **Q2 2020;** **n=4,277,148), n (%)**			
	≤20	742,967 (17.4)	
	21-30	447,669 (10.5)	
	31-40	511,062 (11.9)	
	41-50	478,659 (11.2)	
	51-60	717,920 (16.8)	
	61-70	576,561 (13.5)	
	71-80	429,612 (10)	
	≥81	372,698 (8.7)	
**Employment status** **(Q2 2020;** **n=4,229,406), n (%)**			
	Employed persons (employees/workers, self-employed persons)	2,047,604 (48.4)	
	Not employed persons (unemployed, social welfare recipients, emigrants, refugees, students)	967,533 (22.9)	
	Retired persons	1,214,269 (28.7)	
**Region type of the place of residence (Q2 2020; n=4,277,139), n (%)**			
	Urban regions	2,295,715 (53.7)	
	Mixed regions	1,379,654 (32.2)	
	Rural regions	601,770 (14.1)	

^a^Q2: second quarter.

^b^Q4: fourth quarter.

^c^Q1: first quarter.

**Table 2:** Association of Statutory Health Insurance Physicians (ASHIP) data.

Data type			Data
Source			All SHI^a^ physicians and psychotherapists in 4 ASHIP regions: Berlin, Schleswig-Holstein, Mecklenburg-Western Pomerania, Westphalia-Lippe
Regions			4 of 17 regions in Germany: Berlin, Schleswig-Holstein, Mecklenburg-Western Pomerania, Westphalia-Lippe
Time period			Q2^b^ 2017 until the end of 2020
Exclusion criteria			Specialist groups that are not allowed to provide video consultations (eg, laboratory physicians)
**Physicians (Q2 2017-Q4^c^ 2020)**			
	Minimum (Q2 2017), n	32,128	
	Maximum (Q4 2020), n	34,127	
	Q2 2020, n	33,758	
	Average	33,231	
**Gender** **(Q2 2020;** **n=33,758), n (%)**			
	Female (including diverse)	14,930 (44.2)	
	Male	18,828 (55.8)	
**Age (years;** **Q2 2020;** **n=33,758), n (%)**			
	≤40	2516 (7.5)	
	41-50	7940 (23.5)	
	51-60	12,840 (38)	
	≥61	10,462 (31)	
**Aggregated specialist groups** **(Q2 2020;** **n=33,765), n (%)**			
	Primary care	11,988 (35.5)	
	Psychotherapy, psychiatry, neurology	8663 (25.7)	
	Gynecology	2492 (7.4)	
	Orthopedics	1459 (4.3)	
	Surgery	1391 (4.1)	
	Dermatology	791 (2.3)	
	Otorhinolaryngology and phoniatrics	947 (2.8)	
	Other specialist groups	6034 (17.9)	
**Type of practice (Q2 2020; n=33,782^d^), n (%)**			
	Individual practice	17,904 (53)	
	Joint practices (“Berufsausübungsgemeinschaft” and “Praxisgemeinschaft“)	10,738 (31.8)	
	Medical care center (“Medizinisches Versorgungszentrum [MVZ]“)	3585 (10.6)	
	Hospital physicians authorized for outpatient care	1555 (4.6)	
**Region type of the location of the physician’s office** **(Q2 2020;** **n=33,757), n (%)**			
	Urban regions	19,128 (56.7)	
	Mixed regions	10,086 (29.9)	
	Rural regions	4543 (13.5)	

^a^SHI: Social Health Insurance.

^b^Q2: second quarter.

^c^Q4: fourth quarter.

^d^The sample size is higher than that of Q2 2020 overall, because physicians may be assigned to multiple practice types.

Finally, it is important to emphasize that, depending on which data set is used for analysis (SHIF data or ASHIP data), either the entire outpatient medical care of all SHI-insured persons with residence in the 4 regions is taken into account (ASHIP data) or only the medical care of those SHI-insured persons who are insured with 1 of the 3 SHIFs and live in the 4 regions (SHIF data) is taken into account.

### Data Analysis

The German Federal Data Protection Act defines the principle of data avoidance and data minimization. Accordingly, the aim of the evaluation must always be taken into account when selecting and designing data processing systems. In light of this, a 2-step evaluation was carried out in this analysis: in the first step, a preliminary analysis of the individual data was carried out by the SHIFs and ASHIPs, and, in the second step, the aggregated data were merged and finally evaluated. This was to ensure that the evaluating body (University of Duisburg-Essen) only received the information necessary to answer the research questions.

#### Step 1: Preparatory Work and Preliminary Analysis of Personal Data at the SHIFs

In preparation for the data analysis, an evaluation concept and a data protection concept were first developed. Data forwarding agreements were also concluded bilaterally between the evaluating body and the 7 data providers for the forwarding of the aggregated data.

The developed evaluation concept comprised tables to be completed by all data providers on the use of video consultations, differentiated according to various characteristics of the insured (age, gender, employment status, residence or type of region) and of the physicians and psychotherapists (age, gender, specialist group, type of operation and employment, type of region of the practice location). This information was collected for users of video consultations as well as nonusers of video consultations. The frequency of video consultation use can be identified using the FSP 01450 (technical surcharge for video consultations), as this can be billed for every video contact. A limitation in the use of this FSP is the fact that there is a maximum billing quota for this FSP. In this respect, the use of this FSP requires the assumption that physicians will continue to use FSP 01450 even if they have already exhausted their billing quota per physician and quarter.

Due to the aforementioned (partial) flat-rate billing in outpatient medical care in Germany, which also includes video consultations, an evaluation of the specific services performed in video consultations is only possible to a limited extent. However, it is possible to evaluate certain individual services that are provided during video consultations that can be billed separately. This is possible because the fee schedule items are provided with a standardized national label if they are performed by video. A list of these services has been published by the National Association of Statutory Health Insurance Physicians [10].

Based on this, the preliminary analyses of the individual data were carried out by the SHIFs and ASHIPs.

On the basis of the data forwarding agreements, these were transmitted securely by each data supplier to the evaluating body.

#### Step 2: Merging and Final Evaluation of the Anonymized and Aggregated Data at the Evaluating Body

The aggregated data for each data supplier were initially merged by the evaluating body so that all further evaluations were based on the data from all SHIFs or all ASHIPs. Since the data were not provided at the individual level, the analysis of the aggregated data (only available as cross tables) was performed using Excel (version 16.0; Microsoft Corp).

The analyses included a comparison of video consultation users (VC users) versus nonusers, differentiated according to subgroups (insured: age group, gender, employment situation; physicians and psychotherapists: specialist group, age group, gender, type of operation, and employment). Furthermore, the association between the place of residence or type of region of the practice location and the use of video consultation was examined. To identify significant differences between subgroups, a significance level of *P*<.05 was set. Since the analysis was based on aggregated data (in the form of cross-tabulations, nominally scaled data), chi-square tests were used. Analyses based on individual and microdata (eg, regression analyses) were therefore excluded. However, this approach was chosen for reasons of data economy and because the data analysis was only a preparatory step in the overall project.

### Ethical Considerations

The study was granted ethical approval by the Ethics Committee of the Medical Faculty of the University of Duisburg-Essen on September 27, 2022 (reference: 21-10283-BO). As the University of Duisburg-Essen only had access to aggregated data, there was no possibility of participant identification. 

## Results

### Development of the Use of Video Consultations Over Time

From 2017 to 2019, video consultations played almost no role in outpatient medical care in Germany (quarters 2 and 3 [Q2 and Q3] 2017: 0; Q4 2017-Q3 2019: <300 video consultations; Q4 2019: 718 video consultations). Since the start of the COVID-19 pandemic, the use of video consultations has increased substantially. The billing frequency of FSP 01450 showed a huge increase in the first quarter of 2020 in all regions considered; this continued in the second quarter of 2020. This means that the number of video consultations carried out within 1 quarter increased approximately 6-fold from 44,680 (Q1 2020) to 271,483 (Q2 2020). The second quarter of 2020 covers the largest period of the first lockdown during the COVID-19 pandemic. In the summer months (Q3 2020), the use of video consultations decreased (109,683, which corresponds to only 40.4% [109,683/271,483] of the amount in Q2 2020), although the number of video consultations carried out then rose sharply again in the fourth quarter (192,405, which corresponds to 70.9% [192,405/271,483] of the amount in Q2 2020). This clearly shows that the use of video consultations not only increased sharply during the COVID-19 pandemic but also largely reflected the development of COVID-19 infections. In the period from April 2017 to the end of 2020, a total of 620,970 video consultations were held in the relevant regions in Germany.

When looking at the absolute figures, however, it should be noted that physician contacts with video consultations still only accounted for a fraction of all contacts of this type. If we look at the SHIF data and examine which of the insured persons included had at least one physician contact (“users”; Q2 2020: 4,277,149/6,318,851, 67.7%), the proportion of those who had a video consultation was extremely low, with a maximum of 1% (43,139/4,227,149) in the second quarter of 2020, 0.3% (13,543/4,227,149) in the first quarter of 2020, 0.4% (19,989/4,227,149) in the third quarter of 2020, and 0.8% (34,557/4,227,149) in the fourth quarter of 2020.

By examining the number of video consultations used per VC user, we see that, in the second quarter of 2020, 50% of VC users (21,569/43,139) had a maximum of 1 video consultation. The mean value per VC user was significantly higher, at 2.5 video consultations, which is due to the fact that some VC users made use of a large number of video consultations; the maximum here was 38 video consultations in the second quarter of 2020.

### Characteristics of VC User Groups (Patients)

#### Overview of Analysis of VC User Groups

In the following sections, the results of the comparison between VC users and nonusers in terms of sociodemographic information are presented in order to investigate whether they differed systematically from each other. In addition, the association between the patient’s place of residence and the use of video consultations was also analyzed. The underlying population for all analyses was the insured who had at least one outpatient medical contact in the second quarter of 2020, because only they were faced with the decision of a video consultation or face-to-face contact. The second quarter of 2020 was selected for analysis because, as previously described, the number of video consultations was highest during this period, allowing for subgroup analyses despite the absolute small number of VC users.

#### Differences by Age Group

Table 3 shows, based on the example of the second quarter of 2020, that video consultations were mainly used by young to middle-aged insured persons (20-50 years old). The largest group was those aged 31 years to 40 years, with a share of 1.7% (9378/563,715) of all insured in this age group, with at least one outpatient contact with a doctor (users). The correlation was statistically significant (χ^2^_7_=9903.2, *P*<.001).

**Table 3:** Insured with at least one video consultation (VC) by age group (second quarter 2020), based on Social Health Insurance Fund data (χ27=9903.2, *P*<.001).

≥1 VC	Age (years), n (%)							
	≤20 (n=840,922)	21-30 (n=506,117)	31-40 (n=563,715)	41-50 (n=505,860)	51-60 (n=718,517)	61-70 (n=551,065)	71-80 (n=397,565)	≥81 (n=334,520)
Yes	6600 (0.8)	7287 (1.4)	9378 (1.7)	6304 (1.3)	7140 (1)	3036 (0.6)	1589 (0.4)	1805 (0.5)
No	834,322 (99.2)	498,830 (98.6)	554,337 (98.3)	499,556 (98.7)	711,377 (99)	548,029 (99.4)	395,976 (99.6)	332,715 (99.5)

#### Differences by Gender

Table 4 shows that video consultations were used disproportionately more often by women in the second quarter of 2020. The correlation was statistically significant (χ^2^_1_=1533.8, *P*<.001).

**Table 4:** Insured with at least one video consultation (VC) by gender (second quarter 2020), based on Social Health Insurance Fund data (χ21=1533.8, *P*<.001).

≥1 VC	Women (n=2,369,554), n (%)	Men (n=1,907,594), n (%)
Yes	27,922 (1.2)	15,217 (0.8)
No	2,341,632 (98.8)	1,892,377 (99.2)

#### Employment Status

Table 5 shows that, for the second quarter of 2020, video consultations were used disproportionately often by employed people than people not in employment and retired persons (χ^2^_2_=3566.2, *P*<.001).

**Table 5:** Insured with at least one video consultation (VC) by employment status (second quarter 2020), based on Social Health Insurance Fund data (χ22=3566.2, *P*<.001).

≥1 VC	Employed persons (employees/workers, self-employed persons; n=2,150,544), n (%)	Not employed persons (unemployed, social welfare recipients, emigrants, refugees, students; n=1,096,458), n (%)	Retired persons (n=1,123,538), n (%)
Yes	25,729 (1.2)	8737 (0.8)	7157 (0.6)
No	2,124,815 (98.8)	1,087,721 (99.2)	1,116,381 (99.4)

#### Place of Residence of the Insured

Table 6 shows that video consultations were used disproportionately more often by the insured living in urban regions in the second quarter of 2020 (χ^2^_2_=3746.2, *P*<.001). Considering that a central reason for the introduction of video consultations was to improve access in rural areas, this is striking.

**Table 6:** Insured with at least one video consultation (VC) by place of residence (second quarter 2020), based on Social Health Insurance Fund data (χ22=3746.2, *P*<.001).

≥1 VC	Urban regions (n=2,380,202), n (%)	Mixed regions (n=1,428,651), n (%)	Rural regions (n=609,420), n (%)
Yes	29,184 (1.2)	10,878 (0.8)	3077 (0.5)
No	2,351,018 (98.8)	1,417,773 (99.2)	606,343 (99.5)

In addition, we investigated how video consultations were integrated into the overall treatment of a patient during the quarter; was this done exclusively via video, or was the video consultation only used as a supplement? The exclusive use of video consultations for the treatment of a patient by a physician in a quarter can be identified by a mandatory flag, which leads to a deduction from the physician's quarterly flat rate. Overall, video consultations were used as a supplement in 80% (34,525/43,139) of cases. If this is broken down by region, major differences become apparent. VC users in rural regions used them disproportionately more often exclusively (2001/3077, 65%) compared with those living in urban (4508/29,284, 15.4%) or mixed (2105/10,878, 19.4%) regions (see Table 7).

**Table 7:** Exclusive or only supplementary use of video consultations (VCs) by place of residence (second quarter 2020), based on Social Health Insurance Fund data (χ22=4285.8, *P*<.001).

Type of VC use	Urban regions (n=29,184), n (%)	Mixed regions (n=10,878), n (%)	Rural regions (n=3077), n (%)	Total (n=43,139), n (%)
VC exclusively	4508 (15.4)	2105 (19.4)	2001 (65)	8614 (20)
VC supplementary	24,676 (84.6)	8773 (80.6)	1076 (35)	34,525 (80)

Video consultations were therefore used less frequently in rural regions, but when they are used in these regions, in 65% (2001/3077) of cases, there was no further personal doctor-patient contact during the quarter.

### Characteristics of the Provider Groups (Physicians and Psychotherapists)

#### Overview of Analysis of the Provider Groups

In the following sections, the results of the comparisons between video consultation providers (VC providers) and nonproviders are presented based on ASHIP data with regard to demographic information, information on their specialist group and practice, and employment type. In addition, we analyzed whether there was an association between the location of the medical practice and the availability of video consultations. The comparison was made on the basis of treatment cases provided by the respective physicians and psychotherapists, with or without the use of video consultations.

#### Differences by Age Group

Table 8 shows that, for the second quarter of 2020, physicians and psychotherapists in younger age groups had more treatment cases with video consultations than older physicians and psychotherapists. The largest group in relative numbers was those younger than 40 years (12,680/1,235,393, 1%), while only 0.4% (22,766/6,374,630) of treatment cases with video consultations were performed among those older than 60 years. The correlation was statistically significant (χ^2^_3_=11,338.2, *P*<.001).

**Table 8:** Treatment cases with and without video consultations (VCs) by physicians or psychotherapists and their age (second quarter 2020), based on Association of Statutory Health Insurance Physicians data (χ23=11,338.2, *P*<.001).

Use of VC	Age (years), n (%)			
	≤40 (n=1,235,393)	41-50 (n=5,480,427)	51-60 (n=10,390,058)	≥61 (n=6,374,630)
With VC	12,680 (1)	28,287 (0.5)	42,208 (0.4)	22,766 (0.4)
Without VC	1,222,713 (99)	5,452,140 (99.5)	10,347,850 (99.6)	6,351,864 (99.6)

#### Differences by Gender

The comparison of service providers by gender showed that female physicians or psychotherapists used video consultations with treatment cases slightly more frequently than men (see Table 9).

**Table 9:** Treatment cases with or without video consultations (VCs) by physicians or psychotherapists and their gender (second quarter 2020), based on Association of Statutory Health Insurance Physicians data (χ21=2492.6, *P*<.001).

Use of VC	Women (n=9,053,887), n (%)	Men (n=13,274,371), n (%)
With VC	51,677 (0.6)	55,979 (0.4)
Without VC	9,002,210 (99.4)	13,218,392 (99.6)

### Information on the Specialist Group

A comparative analysis of the professional groups showed that physicians and psychotherapists working in the field of psychotherapy (for both adults and children) were the most likely to use video consultations. Physicians working in psychotherapy and psychological psychotherapists used video consultations with 15.9% (44,808/282,530) of their treatment cases in the second quarter of 2020, followed by psychotherapists for children, who used video consultations with approximately 10% (10,828/113,293, 9.6%) of their treatment cases. Although the absolute number of treatment cases with video consultations among general practitioners was very high compared with other specialist groups (29,600 compared with 10,828 among psychotherapists for children), their share of video consultations with all treatment cases in this specialist group was very low, at 0.3% (29,600/9,837,118). This is caused by the high number of general practitioners and treatment cases in this group.

#### Type of Practice

Table 10 shows that physicians or psychotherapists in individual practices (73,494/11,089,465, 0.7% of treatment cases) used video consultations 2.4 times more frequently than those in joint practices (23,251/8,453,284, 0.3% of cases); this includes different forms of joint practices.

**Table 10:** Treatment cases with or without video consultations (VCs) by physicians or psychotherapists and their type of practice (second quarter 2020), based on Association of Statutory Health Insurance Physicians data (χ22=14,990.5, *P*<.001).

Use of VC	Individual practice (n=11,162,959), n (%)	Joint practices (n=8,476,535), n (%)	Medical care center (n=2,485,753), n (%)
With VC	73,494 (0.7)	23,251 (0.3)	10,423 (0.4)
Without VC	11,089,465 (99.3)	8,453,284 (99.7)	2,475,330 (99.6)

#### Location of the Physician’s Office

As with the situation for insured persons, Table 11 shows that physicians and psychotherapists in urban regions used video consultations 2.3 times more frequently with treatment cases than those in rural regions.

**Table 11:** Treatment cases with or without video consultations (VCs) by physicians or psychotherapists and their practice location (second quarter 2020), based on Social Health Insurance Fund data (χ22=8474.1, *P*<.001).

Use of VC	Urban regions (n=12,153,573)	Mixed regions (n=6,870,939)	Rural regions (n=3,303,069)
With VC	72,982 (0.6)	26,136 (0.4)	8538 (0.3)
Without VC	12,080,591 (99.4)	6,844,803 (99.6)	3,294,531 (99.7)

### Services and Diagnoses Associated With Video Consultations

In 2020, a total of 715,104 services were identified as having been provided and billed as part of video consultations. Approximately 55% (392,380/715,104, 54.9%) of these were claimable individual psychotherapy services, of which 54% (211,788/392,380) were behavioral psychotherapy, 30.3% (118,986/392,380) were depth psychology psychotherapy, and 15.7% (61,606/392,380) were analytic psychotherapy. Of the 715,104 services provided via video consultation, 30% (214,411/715,104) were counseling services, and about 15% (106,580/715,104, 14.9%) were other psychotherapeutic services. A more detailed analysis showed that the majority of counseling services were psychotherapeutic consultations (168,388/214,411, 78.5%) and only a few were primary care consultations (40,418/214,411, 18.9%).

The analysis of diagnoses (according to the International Statistical Classification of Diseases and Related Health Problems, 10th revision, German Modification) in treatment cases (quarterly reference) with contacts that took place exclusively via video consultation showed a similar picture. The top 10 diagnoses in 2020 included, in particular, diagnoses related to mental and behavioral disorders: reactions to severe stress and adjustment disorders (ICD code F43), depressive episode (F32), other anxiety disorders (F41), recurrent depressive disorder (F33), somatoform disorders (F45), and phobic disorders (F40). In addition, the top 10 most frequently coded diagnoses included back pain (M54), essential (primary) hypertension (I10), and COVID-19 pandemic–related indications such as acute upper respiratory tract infection (J06).

## Discussion

### Principal Findings and Interpretation of Underlying Reasons

In the years 2017 to 2019, almost no video consultations were used in outpatient medical care in the German health care system. This changed significantly with the onset of the COVID-19 pandemic: In the first and second quarters of 2020, there was a sharp increase in the use of video consultations in all regions analyzed. However, even in the second quarter, the proportion of the insured with at least one video consultation was extremely low, at a maximum of 1%. There was a decline from the second to the third quarter, with a further significant increase in the fourth quarter—a trend in use that is in line with the COVID-19 infection rate.

Video consultations were used significantly more often by younger insured persons, women, employed persons, and the insured living in urban areas. These findings are in line with the results of other studies stating that the younger and employed generation as well as people living in urban environments are more interested in using video consultations than older age groups or people residing in rural areas [3]. Another reason could be that physicians act as gatekeepers and preselect patients, assuming that younger people and people from urban areas with presumably better internet access are more suitable for video consultations [11]. The picture is similar for physicians and psychotherapists, with younger age groups and physicians and psychotherapists with an urban-based practice also using video consultations more frequently. In order to promote the sustainable use of video consultations, it is important to identify the key barriers for those groups. A lack of technical requirements has often been cited as a key barrier for patients as well as physicians and psychotherapists [12-14]. Physicians were also concerned about negative effects on the doctor-patient relationship. In addition, some believed that the lack of physical examination methods could more easily lead to treatment errors or misdiagnosis. Concerns were also expressed about privacy and data security [11,15].

There was no evidence of increased use in structurally disadvantaged rural areas, an often-cited potential for video consultations [3]. A number of hypotheses may be posited to explain the underlying reasons for the observed phenomenon. For instance, the comparatively poorer broadband infrastructure in rural areas or a different age structure could be identified as potential explanatory factors. The population in rural regions is, on average, older and therefore potentially less digitally proficient than that in urban regions. In addition, people from urban regions tend to use psychotherapy, which is the predominant use case for video consultations, more often than people from rural regions [5].

Psychotherapists and physicians working in a psychotherapy specialty (both for adults and children) were, by far, the most frequent users of video consultations. This was also reflected in the analysis of services and diagnoses in video consultations. The reason for this may be the nature of the services in this area, which are primarily what is known as “talking medicine.” Somatic specialists generally rely on physical examinations, blood samples, for example, as a supplement.

Video consultations are primarily used as an adjunct to care and rarely occur without further doctor-patient contact. However, in rural areas, where video consultations are less common, the opposite is true: There is often no face-to-face contact in addition to video consultations. One reason for this could be that these patients used a video consultation with a central platform rather than with their usual physician. On these platforms, personal follow-up contact is usually not possible at all, as physicians from completely different regions of Germany can also offer video consultations there. This may be because their usual physician does not offer video consultations or because there are no physicians in the area with vacancies in the specialty they are looking for. In any case, this is an indicator of a potential lack of care in rural areas.

### Strengths and Limitations

This data analysis was based on data from 3 large German SHIFs with a wide range of enrollees (about 6.1 million insured or approximately 8.4% of all SHI-insured) and 4 ASHIPs (about 33,000 physicians and psychotherapists or 18.4% of all SHI-accredited physicians and psychotherapists in Germany). It is also worth noting that insured persons from different types of SHIFs were considered, so that they do not represent only one type of SHIF (eg, in terms of age and gender). The selection of the 4 regions ensured that both rural and urban enrollees and providers were included.

A first limitation could be that only those with statutory health insurance were included, not those with private health insurance, which accounts for only about 10% of all insured persons in Germany.

The time period included allowed for an analysis of the impact of the COVID-19 pandemic on the use of video consultations. The second limitation is that this period could also have had a biasing effect, for example in the analysis of diagnoses coded in relation to video consultations, given the extremely high coding of respiratory diseases in the second quarter of 2020. A slightly longer data period would also have been desirable to analyze the effects after the pandemic. However, the data period used, in particular the year 2020, was considered sufficient for the analysis of the characteristics of the user groups.

Third, the statistical evaluation was hindered by a number of evident challenges. One principal reason for this was that, for reasons of data minimization, only aggregated data were made available to the evaluator, which significantly constrained the range of analysis options. Consequently, only chi-square analysis and, for example, no regression analyses could be conducted. It should be noted, however, that even minor discrepancies can become statistically significant due to the large sample size. To ensure transparency, the absolute and relative data were consistently provided in the cross tables, allowing readers to assess them independently. Additionally, the study faced a challenge with balancing the proportions, with approximately 1% of the sample using a video consultation and the remaining 99% not using it. This imbalance can complicate the analysis.

Fourth, not all variables are available in health insurance data; for example, information on educational status would have been useful. However, in this mixed methods project, a survey was conducted to ask, for example, about the use of video consultations and the level of education [8]. The findings will be published separately.

Fifth, the generalizability of the results for Germany to other countries may be limited. Aspects such as the slow progress of digitization in Germany, as well as the existence of statutory health insurance and a relatively high density of physicians compared with other countries, could explain the hesitant acceptance of video consultations and may not apply to other countries.

### Conclusion and Potential for Future Research

The current extent of video consultation use remains well below its potential. Additionally, it is notable that the groups for whom it was anticipated that video consultations would offer substantial benefits, such as individuals with limited mobility and those residing in rural areas, currently use video consultations at a significantly lower rate than groups who are currently considered to be well served, including individuals in urban areas and young people.

Unfortunately, initial analyses of a period at the end of the pandemic are concerning. According to these data, the already low uptake rate declined further toward the end of the pandemic. According to a report by BARMER, a German SHIF, monthly uptake in the second quarter of 2022 was more than 50% lower than during the pandemic waves in the second quarter of 2020 and the first quarter of 2021. It appears to have stabilized at a relatively low level [16].

The widespread and lasting use of video consultations will only succeed if the potential user groups accept this form of service provision and recognize its advantages. Further analyses should therefore investigate the preferences of user groups for the use of video consultations. Both qualitative (interviews, focus group discussions) and quantitative research approaches (surveys [eg, with preference elicitation methods such as discrete choice experiments]) are suitable for this purpose. As part of this study, a survey was conducted among the insured, physicians, and psychotherapists to identify the primary barriers to the use of video consultations [7]. The findings will be released in a separate publication.

## Abbreviations

ASHIP: Association of Statutory Health Insurance Physicians
FSP: fee schedule position
Q: quarter
SHI: Social Health Insurance
SHIF: Social Health Insurance Fund
VC: video consultation

## Data Availability

The data sets on which the routine data analysis is based are not publicly available for privacy reasons.

## References

[1] (2022). Bericht des Bewertungsausschusses und des ergänzenden Bewertungsausschusses zur telemedizinischen Leistungserbringung im Einheitlichen Bewertungsmaßstab (BT-Drucksache 20/4982). Deutscher Bundestag.

[2] (2022). Consolidated telemedicine implementation guide. World Health Organization.

[3] Waschkau A, Traulsen P, Steinhäuser Jost (2022). Evaluation of synchronous and asynchronous telemedical applications in primary care in rural regions of northern Germany-results and lessons learned from a pilot study. Int J Environ Res Public Health.

[4] (2018). Unterrichtung durch die Bundesregierung - Bericht des Bewertungsausschusses zur Überprüfung des Einheitlichen Bewertungsmaßstabes auf die Möglichkeit zur ambulanten telemedizinischen Leistungserbringung (BT-Drucksache 19/0620). Deutscher Bundestag.

[5] Gensorowsky D, Surmann B, Schmidt J, Greiner W (2022). [Level of use and user groups of online video consultations in outpatient medical care: analysis of claims data]. Gesundheitswesen.

[6] Petrick N, Kreuzenbeck CCJ (2023). [Effects of the Covid-19 pandemic on online use of video consultation by general practitioners in Germany - a secondary data analysis of German health insurance data]. Gesundheitswesen.

[7] Kleinschmidt L, Walendzik A, Wasem J, Höfer Klemens, Nauendorf B, Brittner M, Brandenburg P, Aeustergerling A, Schneider U, Wadeck A, Sehlen S, Liersch S, Schwarze K, Schwenke C, Hüer Theresa (2024). Preference-based implementation of video consultations in urban and rural regions in outpatient care in Germany: protocol for a mixed methods study. JMIR Res Protoc.

[8] (2020). Gesetzliche Krankenversicherung. Mitglieder, mitversicherte Angehörige und Krankenstand. Jahresdurchschnitt 2020. Bundesministerium für Gesundheit.

[9] (2020). Federal Medical Register. Kassenärztliche Bundesvereinigung.

[10] (2024). Übersicht Vergütung für Videosprechstunde. Kassenärztliche Bundesvereinigung.

[11] Cowan KE, McKean AJ, Gentry MT, Hilty DM (2019). Barriers to use of telepsychiatry: clinicians as gatekeepers. Mayo Clin Proc.

[12] Thiyagarajan A, Grant C, Griffiths F, Atherton H (2020). Exploring patients' and clinicians' experiences of video consultations in primary care: a systematic scoping review. BJGP Open.

[13] Donaghy E, Atherton H, Hammersley V, McNeilly H, Bikker A, Robbins L, Campbell J, McKinstry B (2019). Acceptability, benefits, and challenges of video consulting: a qualitative study in primary care. Br J Gen Pract.

[14] Christensen LF, Moller AM, Hansen JP, Nielsen CT, Gildberg FA (2020). Patients' and providers' experiences with video consultations used in the treatment of older patients with unipolar depression: a systematic review. J Psychiatr Ment Health Nurs.

[15] Mueller M, Knop M, Niehaves B, Adarkwah CC (2020). Investigating the acceptance of video consultation by patients in rural primary care: empirical comparison of preusers and actual users. JMIR Med Inform.

[16] Mangiapane S, Repschläger U (2023). Five years of video consultations in statutory health care: Has the pandemic led to a breakthrough?. BARMER Gesundheitswesen.

